# Downregulating miR-184 relieves calcium oxalate crystal-mediated renal cell damage via activating the Rap1 signaling pathway

**DOI:** 10.18632/aging.205286

**Published:** 2023-12-27

**Authors:** Mei Han, Donghong Zhang, Junwei Ji, Junli Zhang, Mingyi Qin

**Affiliations:** 1 Department of Emergency, The Fourth Hospital of Hebei Medical University, Shijiazhuang 050000, China; 2 Department of Emergency, The Second Hospital of Hebei Medical University, Shijiazhuang 050000, China; 3 Department of Nursing, The Fourth Hospital of Hebei Medical University, Shijiazhuang 050000, China

**Keywords:** miR-484, inflammation, renal injury, calcium oxalate crystal

## Abstract

Background: Renal calculi are a very prevalent disease with a high incidence. Calcium oxalate (CaOx) is a primary constituent of kidney stones. Our paper probes the regulatory function and mechanism of miR-184 in CaOx-mediated renal cell damage.

Methods: CaOx was used to treat HK2 cells and human podocytes (HPCs) to simulate kidney cell damage. The qRT-PCR technique checked the profiles of miR-184 and IGF1R. The examination of cell proliferation was conducted employing CCK8. TUNEL staining was used to monitor cell apoptosis. Western blot analysis was used to determine the protein profiles of apoptosis-concerned related proteins (including Mcl1, Bcl-XL, and Caspase-3), the NF-κB, Nrf2/HO-1, and Rap1 signaling pathways. ELISA confirmed the levels of the inflammatory factors IL-6, TNF-α, MCP1, and ICAM1. The targeting relationship between miR-184 and IGF1R was validated by dual luciferase assay and RNA immunoprecipitation assay.

Results: Glyoxylate-induced rat kidney stones model and HK2 and HPC cells treated with CaOx demonstrated an increase in the miR-184 profile. Inhibiting miR-184 relieved CaOx-mediated renal cell inflammation, apoptosis and oxidative stress and activated the Rap1 pathway. IGF1R was targeted by miR-184. IGF1R activation by IGF1 attenuated the effects of miR-184 on renal cell damage, and Hippo pathway suppression reversed the inhibitory effect of miR-184 knockdown on renal cell impairment.

Conclusions: miR-184 downregulation activates the Rap1 signaling pathway to ameliorate renal cell damage mediated by CaOx.

## INTRODUCTION

Kidney stones, an old disease shown by the formation of stones in the kidneys, ranks as one of the oldest known and widespread diseases worldwide. Generally, about 10–12% of people in industrialized countries are found with this disease, and the relapse rate reaches 50% in 5–10 years after first being diagnosed for the first time [1, 2]. Calcium oxalate (CaOx) constitutes the bulk of most kidney stones (75-90% of all cases), followed by (75–90%), followed by magnesium ammonium phosphate (struvite) (10–15%), uric acid (3–10%), and cystine (0.5–1%) [3]. The disease can be prevented and controlled by augmenting the intake of fluid and decreasing foods abounding in calcium or oxalic acid, covering meat, spinach, and tofu [4]. To relieve pain in patients with kidney stones, nonsteroidal anti-inflammatory drugs are the first choice. For the recurrence of calcium stones, it’s recommended that thiazide diuretics, potassium citrate, or allopurinol should be prescribed for those patients [5]. Further investigating the underlying mechanism of kidney stones might provide novel references for its treatment.

MicroRNAs (miRNAs) are small noncoding RNAs that can influence messenger RNA translation and degradation to modulate gene expression [6]. Several miRNAs have been confirmed to exert a significant regulatory function in renal cell injury. For instance, miR-20b-3p had a decreased level in the urine of patients with hyperoxaluria. miR-20b-3p shuttled by exosomes originated from adipose-derived stromal cells, can mitigate cell autophagy and inflammation that were induced by oxalate. Besides, miR-20b-3p-enriched exosomes had protective effects in a hyperoxaluria rat model triggered by ethylene glycol [7]. Jiang K et al. revealed that miR-155-5p targets and restrains matrix Gla protein (MGP) expression to boost renal oxidative stress damage caused by oxalate and calcium oxalate monohydrate [8]. miR-184 is a vital member of miRNAs. It has elevated expression in patients with renal carcinoma [9] and serves as a predictive biomarker of cardiac damage [10]. Additionally, miR-184 showed altered expression in 8-month-old Zucker diabetic fatty (ZDF) rats and enhanced renal fibrosis [11]. Nevertheless, we still have no idea about the regulatory function of miR-184 in renal cell damage mediated by calcium oxalate.

The Rap1 signaling pathway, a small GTPase signaling pathway, plays a significant role in various cellular processes including inflammation, apoptosis, and oxidative stress [12–14]. Rap1 signaling pathway interacts with several key signaling pathways, such as the NF-κB pathway [12] and AKT/Nrf-2/HO-1 pathway [15], to regulate gene expression and modulate cellular responses. The Rap1 pathway is pivotal to sustaining cell homeostasis, and its aberrant expression usually pertains to the pathology of multiple diseases [16, 17]. A recent study has suggested that cAMP-Epac-Rap signaling pathway activation exhibits protective effects in a cisplatin-induced renal cell injury model through mediating cell-cell junctions and cell apoptosis [18]. This study supports that Rap1pathway has a role in mediating renal injury.

Here, the authors have found that miR-184 showed significant upregulation in genetic hypercalciuric stone-forming (GHS) rats compared to normal rats as well as in renal cell damage triggered by calcium oxalate. Our further experiments showed that miR-184 expression was upregulated in rat renal calcinosis tissues and that miR-184 inhibition mitigated calcium oxalate-induced damage to kidney cells. Furthermore, we conducted bioinformatic analysis, which revealed that miR-184 potentially targets insulin-like growth factor 1 receptor (IGF1R) and is involved in the Rap1 signaling pathway. This suggests that targeting miR-184 could be a new approach for treating kidney stones by mediating Rap1 signaling pathway. We hope this study provides a novel reference for kidney stones diagnosis and therapy.

## RESULTS

### The profile features of miR-184 in the kidney stones model

**Figure 1:**
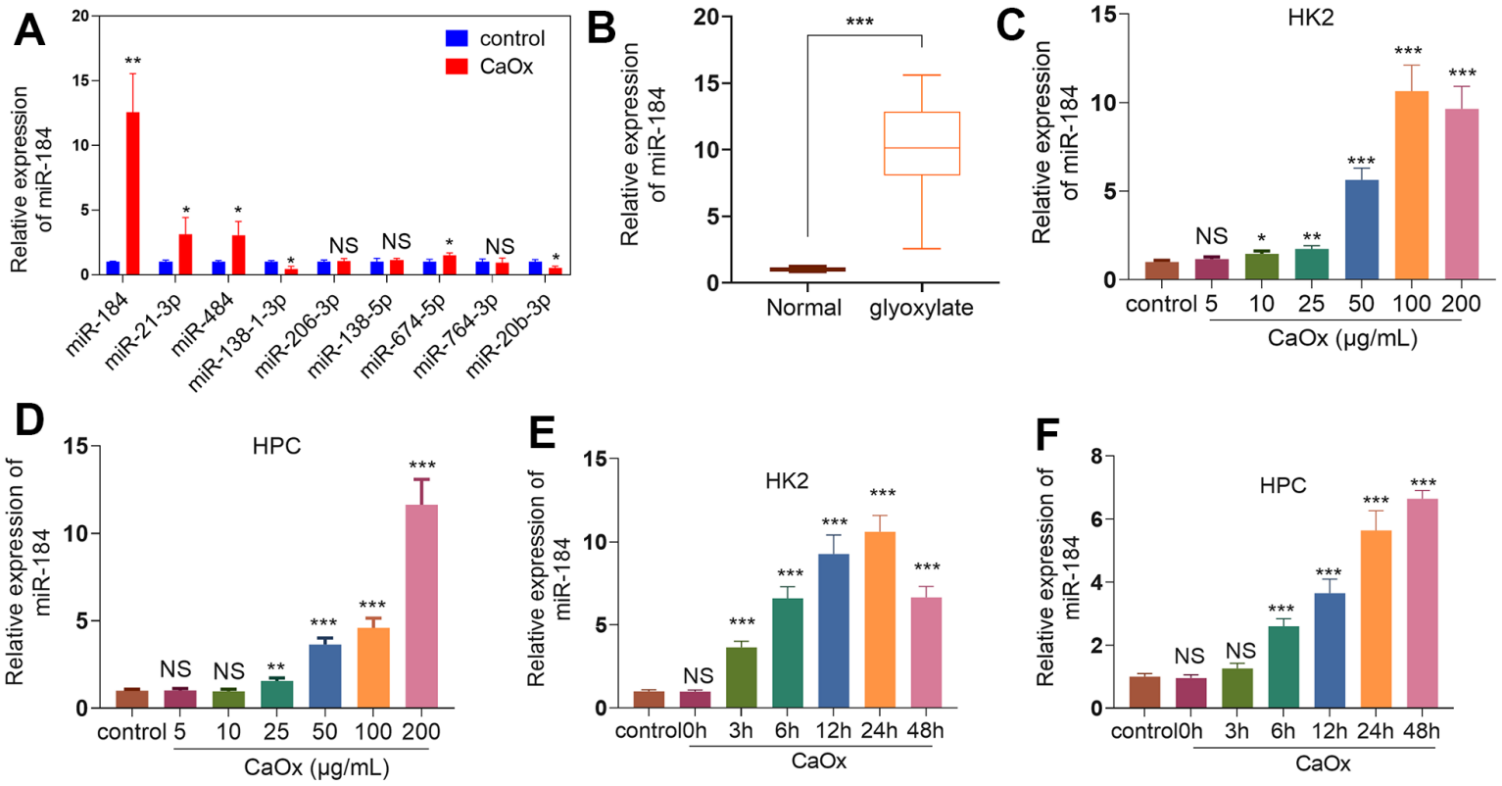
**miR-184 expression features in the kidney stones model.** (**A**) CaOx (100 μg/mL) was applied to treat HK2 cells for 24 hours. The top 10 DE miRNAs were determined by RT-PCR. (**B**) The rat kidney stones model was induced through the intraperitoneal injection of glyoxylate (80 mg/kg/d; 200 μL). qRT-PCR examined miR-184 expression in the kidney tissues of the rats. ***P<0.001 (vs. Normal). N=10. (**C**, **D**) CaOx (5, 10, 25, 50, 100, 200 μg/mL) was applied to treat HK2 cells and HPCs for 24 hours. qRT-PCR analysis of miR-184 expression in HK2 and HPC cells. (**E**, **F**) CaOx (100 μg/mL) was applied to treat HK2 cells and HPCs for 3, 6, 12, 24, 48 hours. qRT-PCR analysis of miR-184 expression in HK2 and HPC cells. NSP>0.05, *P<0.05, **P<0.01, ***P<0.001 (vs. control). N=3.

Differentially expressed miRNAs in genetic hypercalciuric stone-forming (GHS) rats compared to normal rats from the public Gene Expression Omnibus (GEO) database (GSE75541) were analyzed (Table 1). We tested those 10 miRNAs in HK2 cells dealt with CaOx (100 μg/mL). MiR-184 was significantly altered and had the highest fold changes (Figure 1A). In contrast to the normal group, glyoxylate substantially elevated miR-184 expression in the rats’ kidney tissues (Figure 1B). CaOx (10, 25, 50, 100, and 200 μg/mL) considerably enhanced the miR-184 profile in HK2 and HPC cells vis-a-vis the control group (Figure 1C, 1D). Moreover, CaOx also enhanced miR-184 level in a time-dependent manner (Figure 1E, 1F). These findings signified that miR-184 expression was upregulated in both *in vivo* and *in vitro* renal calcinosis models.

**Table 1:** Altered expression of miRNAs genetic hypercalciuric stone-forming (GHS) rats compared to normal rats.

**adj.P.Val**	**P-Value**	**t**	**B**	**logFC**	**miRNA_ID**
0.0276	2.16E-05	12.03	1.442	4.749	rno-miR-184
0.2746	0.000619	6.58	-0.189	1.944	rno-miR-21-3p
0.2746	0.000814	-6.25	-0.368	-3.708	rno-miR-484
0.2746	0.00086	-6.18	-0.405	-3.316	rno-miR-138-1-3p
0.4133	0.001618	-5.47	-0.845	-1.285	rno-miR-206-3p
0.6193	0.00291	-4.86	-1.282	-1.784	rno-miR-138-5p
0.7296	0.006309	4.13	-1.897	0.548	rno-miR-674-5p
0.7296	0.009473	-3.77	-2.234	-0.486	rno-miR-764-3p
0.7296	0.009474	-3.77	-2.234	-0.772	rno-miR-20b-3p

### miR-184 knockdown weakened renal cell damage mediated by CaOx

**Figure 2:**
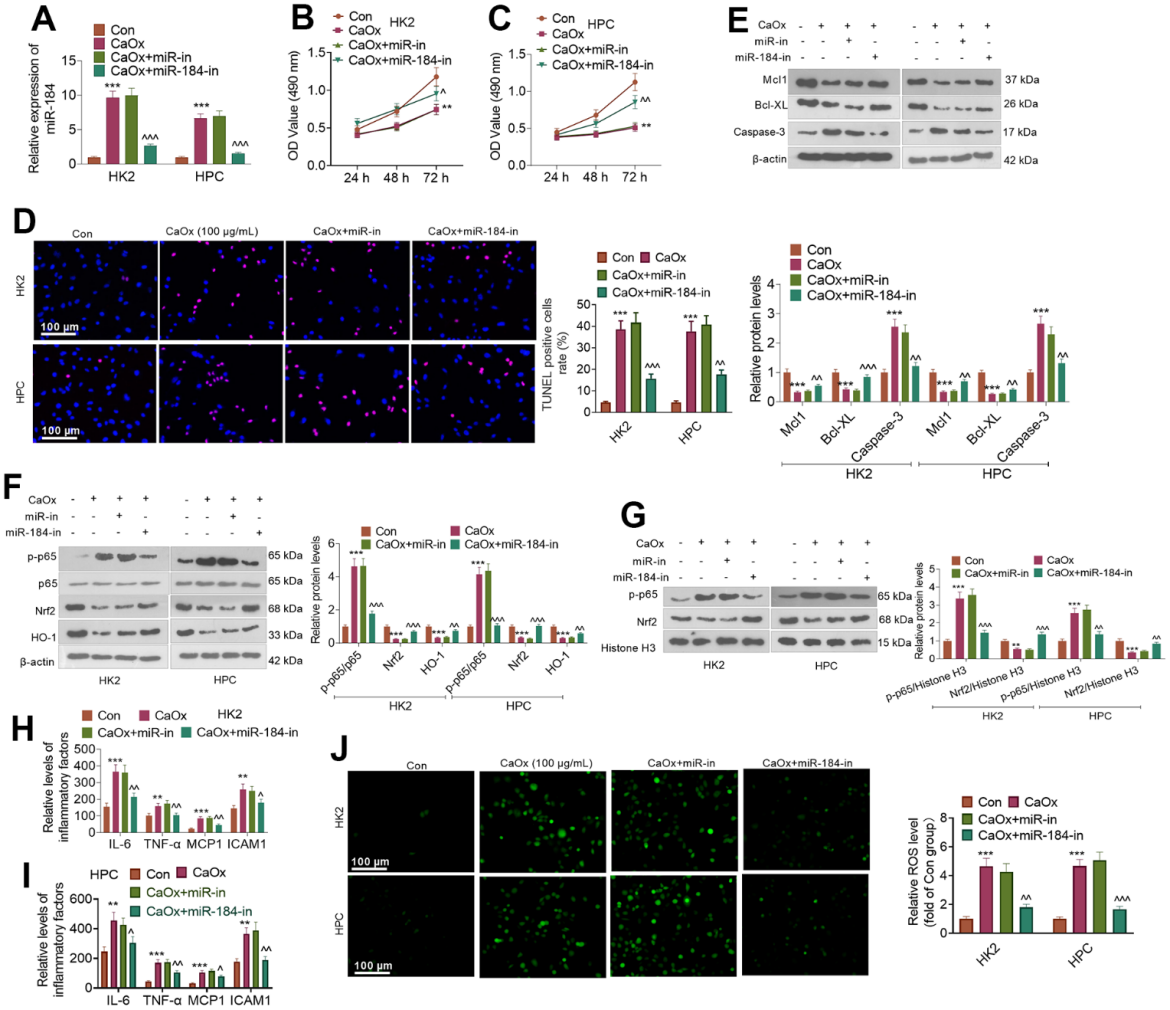
**miR-184 knockdown abated renal cell damage mediated by CaOx.** CaOx (100 μg/mL) was used to treat HK2 and HPC cells, with or without miR-184-in transfection, for 24 hours. (**A**) qRT-PCR analysis of miR-184 expression. (**B**, **C**) Cell viability was examined by CCK8 assay. (**D**) TUNEL staining monitored cell apoptosis. Scale bar=100 μm. (**E**) The profiles of apoptosis-related proteins (Mcl1, Bcl-XL, Caspase-3) were determined by Western blotting. (**F**) The protein profiles of the NF-κB and Nrf2/HO-1 pathways were confirmed through Western blotting. (**G**) The protein levels of NF-κB p65 and Nrf2 in the nucleus were verified through Western blotting. (**H**, **I**) ELISA revealed the levels of the inflammatory factors IL-6, TNF-α, MCP1, and ICAM1. (**J**) Cell immunofluorescence was used to examine the level of ROS. Scale bar=100 μm. **P<0.01, ***P<0.001 (vs. the Con group). *^P<0.05, ^^P<0.01, ^^^P<0.001* (vs. CaOx+miR-in). N=3.

CaOx (100 μg/mL) was applied to treat HK2 and HPC cells treated or untreated with miR-184-in. qRT-PCR indicated that in contrast with the Con group, CaOx prominently bolstered miR-184 expression in HK2 and HPC cells. When compared to the CaOx+miR-in group, miR-184-in transfection evidently reduced the profile of miR-184 (Figure 2A). As indicated by CCK8 assay, CaOx substantially weakened HK2 and HPC viability. When miR-184-in was further transfected, the cell viability was distinctly strengthened (Figure 2B, 2C). TUNEL staining showed that CaOx considerably augmented the proportion of TUNEL-positive HK2 and HPC cells, which was suppressed by miR-184-in (Figure 2D). Western blot analysis ascertained the protein profiles of apoptosis-correlated proteins (Mcl1, Bcl-XL, and Caspase-3,) and of the NF-κB and Nrf2/HO-1 pathways. As shown in Figure 2E, CaOx vigorously repressed the profiles of the anti-apoptosis proteins Mcl1 and Bcl-XL and boosted the protein profiles of the pro-apoptosis protein Caspase-3, but miR-184-in notably reversed this effect. Figure 2F shows that after HK2 cells and HPCs were treated with CaOx, the protein levels of p-p65/p65, Nrf2, and HO-1 were markedly increased. Nevertheless, miR-184-in was transfected on the basis of CaOx, resulting in a marked reduction in their protein profiles. Figure 2G shows that miR-184-in apparently weakened the CaOx-mediated promotion of p-p65 and Nrf2. Given the ELISA results, the levels of the inflammatory factors IL-6, TNF-α, MCP1, and ICAM1 in HK2 cells and HPCs were considerably increased by CaOx (compared with those in the Con group) but markedly decreased by miR-184-in (compared with those in the CaOx+miR-in group) (Figure 2H, 2I). Immunofluorescence indicated that miR-184 inhibited the promoting effect of CaOx on ROS activity (Figure 2J). These discoveries demonstrated that miR-184 knockdown exerted an inhibitory impact on renal cell damage mediated by CaOx.

### miR-184 downregulation activated the Rap1 signaling pathway

**Figure 3:**
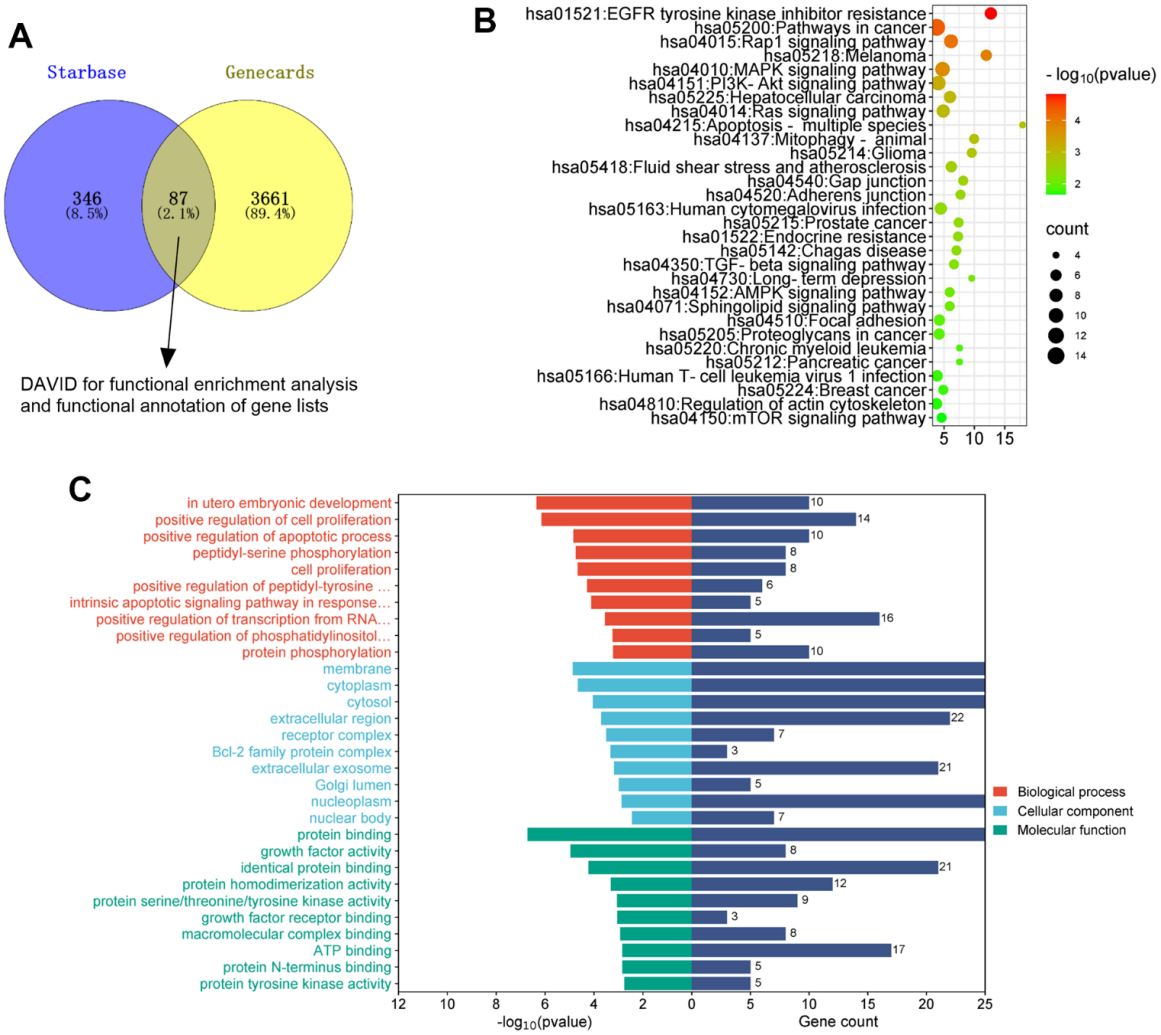
**The analysis of the mechanism of miR-184.** The targets of miR-184 was searched in Starbase. The targets of kidney stones were analyzed in Genecards. (**A**) Venny’s diagram showed that 87 genes were intersected. (**B**, **C**) Functional enrichment analysis was conducted via the online website DAVID (https://david.ncifcrf.gov/home.jsp).

**Figure 4:**
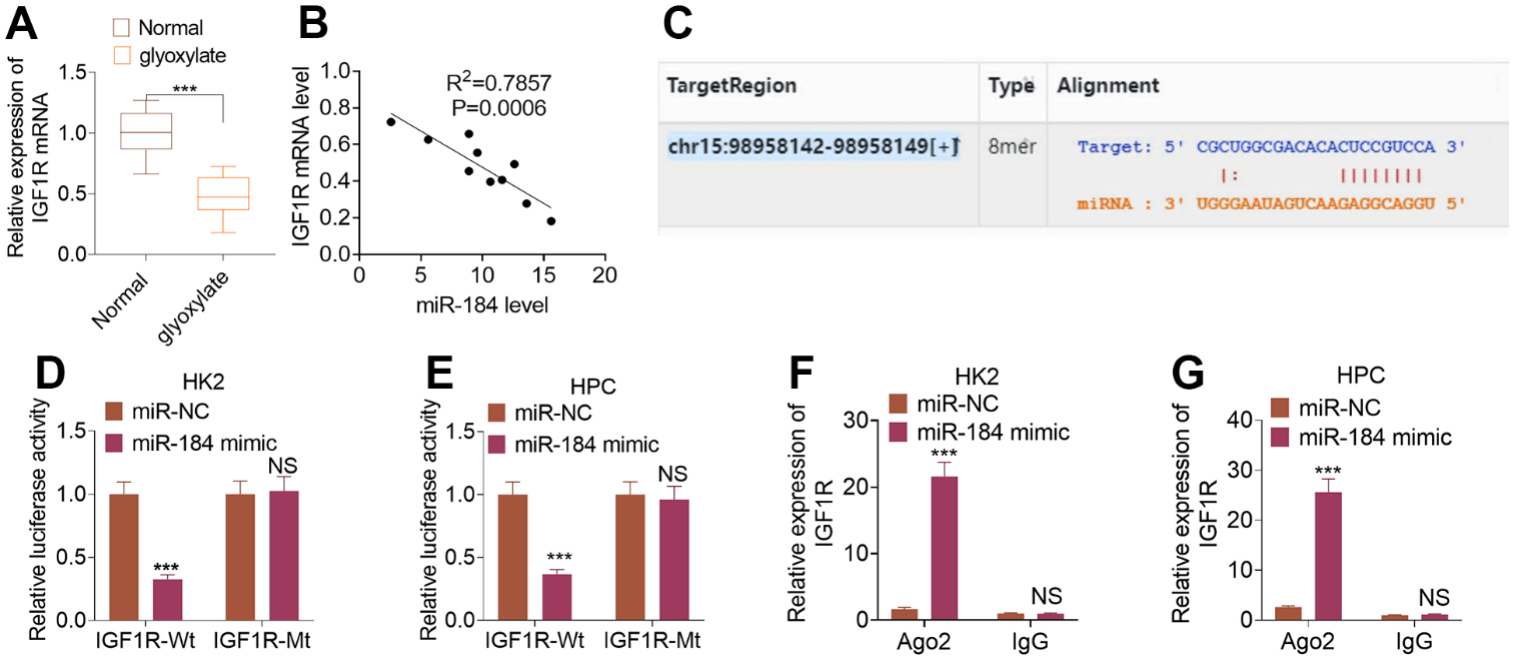
**IGF1R was targeted by miR-184.** The rat renal calcinosis model was induced through the intraperitoneal transfusion of glyoxylate (80 mg/kg/d; 200 μL). (**A**) qRT-PCR was used to ascertain IGF1R expression in rat kidney tissues. ***P<0.001 (vs. Normal). N=10. (**B**) Pearson analysis evaluated the correlation between IGF1R and miR-184 in the renal calcinosis model. (**C**) The complementary base sequence of IGF1R and miR-184 in the Starbase. (**D**–**G**) Dual luciferase and RIP experiments validated the binding correlation between miR-184 and IGF1R. nsP>0.05, **P<0.01, ***P<0.001 (vs. miR-NC). N=3.

**Figure 5:**
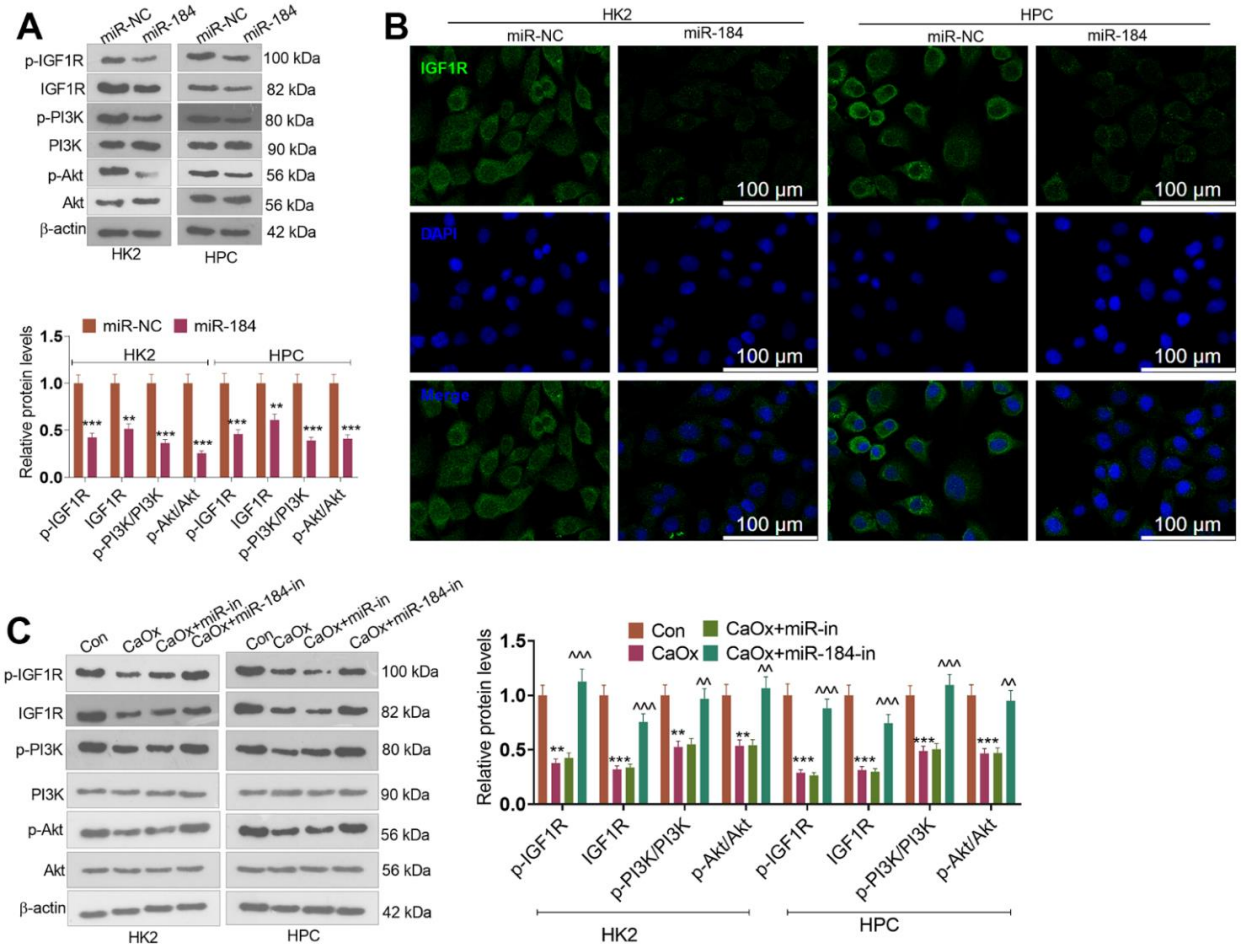
**miR-184 downregulation activated the Rap1 pathway.** miR-NC and miR-184 were used to transfect HK2 cells. miR-in and miR-184-in were used to transfect HPC cells with CaOx (100 μg/mL) for 24 hours of treatment. (**A**) Western blot analysis was used to determine the protein profiles of p-IGF1R, IGF1R, p-PI3K, PI3K, Akt, and p-Akt in the Rap1 pathway. (**B**) Fluorescence was conducted for evaluating IGF1R expression*.* Scale bar=100 μm. (**C**) Western blot analysis was used to determine the protein profiles of p-IGF1R, IGF1R, p-PI3K, PI3K, Akt, and p-Akt in the Rap1 pathway. **P<0.01, ***P<0.001 (vs. Con). ^*^P<0.01, ^^^P<0.001* (vs. CaOx+miR-in). N=3.

To uncover the downstream of miR-184, we predicted the targets of miR-184 via Starbase (https://rnasysu.com/encori/index.php). The targets of kidney stones were analyzed in Genecards (https://www.genecards.org/). Venny’s diagram showed that 87 genes were intersected (Figure 3A). Functional enrichment analysis was conducted via the online website DAVID (https://david.ncifcrf.gov/home.jsp). It was found that miR-184 potentially regulates the Rap1 signaling pathway (Figure 3B) and affects many cellular functions (Figure 3C). IGF1R was among the target genes of miR-184. In the rat kidney stones model, IGF1R was significantly downregulated (Figure 4A) and had a negative relationship with miR-184 (Figure 4B). The binding relationship between miR-184 and IGF1R was shown in Figure 4C. Dual luciferase and RIP assays verified the binding correlation between miR-184 and IGF1R. Dual luciferase assays revealed that in contrast with the miR-NC group, miR-184 mimics prominently hampered IGF1R-Wt expression in HK2 and HPC cells but basically displayed no impact on IGF1R-Mt (Figure 4D, 4E). RIP revealed that in comparison with the miR-NC group, miR-184 mimics greatly facilitated the recruitment of IGF1R mRNA by Ago2 antibody, but their influence on IgG antibody manifested no significant differences (Figure 4F, 4G). Moreover, miR-NC or miR-184 mimics were transfected into HK2 cells. miR-in and miR-184-in were transfected into HPC cells with CaOx (100 μg/mL) adopted for treatment. The protein profiles of IGF1R and PI3K-Akt in the Rap1 pathway were determined via Western blotting. As reflected by Figure 5A, 5B, in contrast with the miR-NC group, miR-184 evidently curbed the profiles of p-IGF1R, IGF1R, p-PI3K, and p-Akt in HK2 and HPC cells. In accordance with Figure 5C, CaOx distinctly restricted the profiles of p-IGF1R, IGF1R, p-PI3K, and p-Akt in HK2 and HPC cells, which were boosted by miR-184-in (in contrast with the CaOx+miR-in group). These outcomes revealed that miR-184 downregulation activated the profile of the Rap1 pathway.

### IGF1R activation attenuated miR-184-mediated renal cell impairment

**Figure 6:**
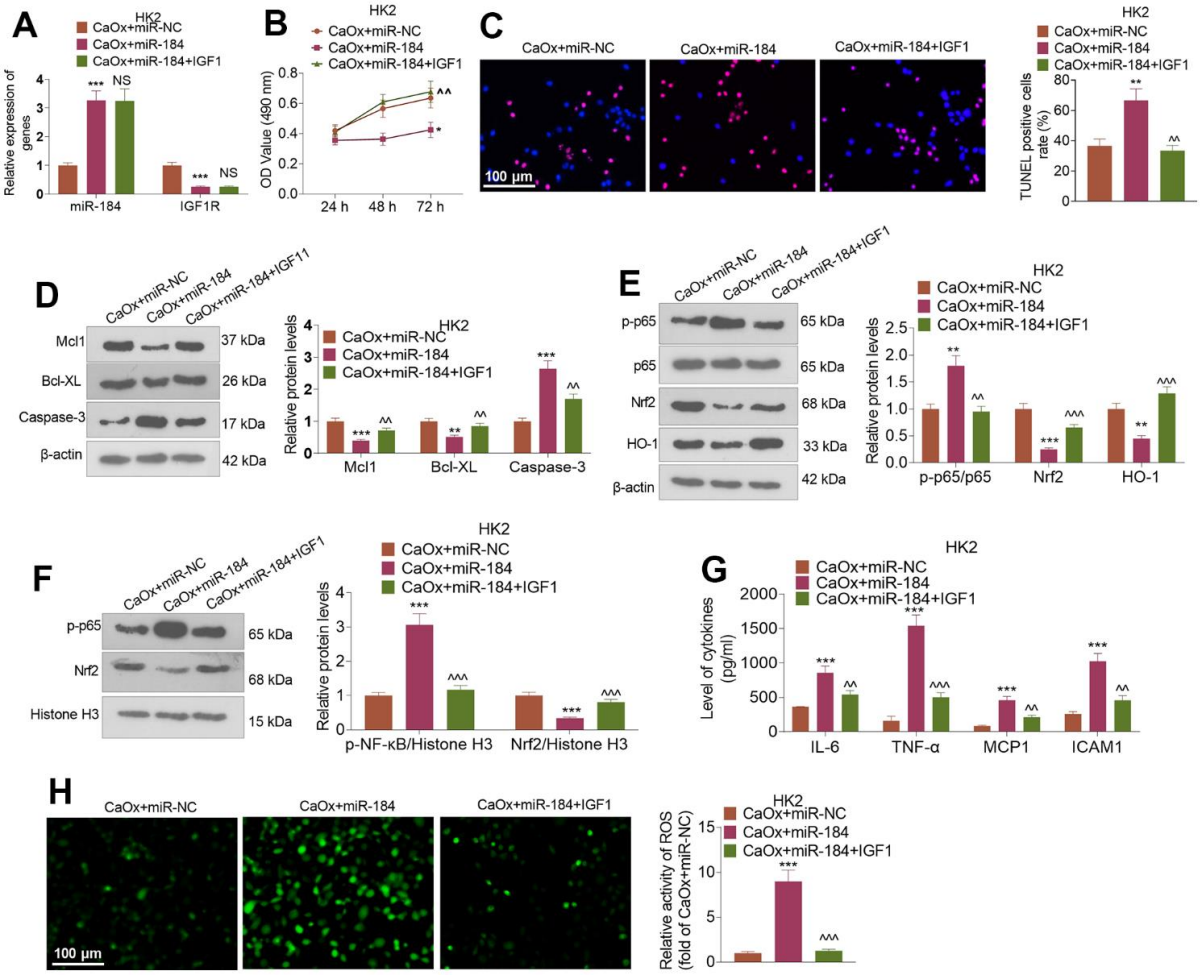
**IGF1 treatment attenuated miR-184-mediated renal cell damage.** CaOx (100 μg/mL) was used to treat HK2 cells transfected with miR-184 mimics and IGF1 (100ng/ml) treatment for 24 hours. (**A**) qRT-PCR was used to examine the profiles of miR-184 and IGF1R. (**B**) CCK8 assay was used to check cell viability. (**C**) TUNEL staining monitored cell apoptosis. Scale bar=100 μm. (**D**) Western blot analysis was used to determine the profiles of apoptosis-related proteins. (**E**, **F**) Western blot analysis was used to determine the profiles of the NF-κB and Nrf2/HO-1 pathways. (**G**) ELISA revealed the levels of the inflammatory cytokines IL-6, TNF-α, MCP1, and ICAM1. (**H**) Immunofluorescence evaluated the activity of ROS. Scale bar=100 μm. *P<0.05, **P<0.01, ***P<0.001 (vs. CaOx+miR-NC). *^^P<0.01*, *^^^P<0.001* (vs. CaOx+miR-184). N=3.

CaOx (100 μg/mL) and/or IGF1 (100 ng/ml) was utilized to treat HK2 cells transfected with miR-184 mimics. qRT-PCR indicated that in contrast to the CaOx+miR-NC group, miR-184 overexpression evidently suppressed IGF1R expression, whereas IGF1 treatment (compared to the CaOx+miR-184 group) did not substantially influence miR-184 expression (Figure 6A). CCK8 assay showed that miR-184 overexpression distinctly hindered HK2 cell viability, whereas IGF1 treatment enhanced their proliferation (Figure 6B). TUNEL staining evaluated HK2 cell apoptosis, signifying that miR-184 overexpression apparently bolstered HK2 cell apoptosis, while IGF1 treatment contributed to the opposite outcome (Figure 6C). As denoted by Western blot, miR-184 overexpression resulted in a decrease in the profiles of the anti-apoptotic proteins Mcl1 and Bcl-XL and an increase in the profiles of the pro-apoptotic proteins Caspase-3 and Caspase-8. When IGF1 treatment was performed, Mcl1 and Bcl-XL expression increased, while Caspase-3 expression decreased (Figure 6D). As presented in Figure 6E, 6F, IGF1 treatment substantially weakened the promoting function of miR-184 overexpression in the p-NF-κB p65 and Nrf2/HO-1 pathways. ELISA revealed that miR-184 overexpression dramatically enhanced the levels of the inflammatory cytokines IL-6, TNF-α, MCP1, and ICAM1, while IGF1 treatment gave rise to the reverse phenomenon (Figure 6G). Immunofluorescence showed that IGF1 treatment considerably dampened the promoting impact of miR-184 overexpression on ROS activity (Figure 6H). These findings revealed that IGF1 treatment abated miR-184-mediated damage-promotive function in renal cells.

### IGF1R inhibition reversed the protective function of miR-184 knockdown in renal cells

**Figure 7:**
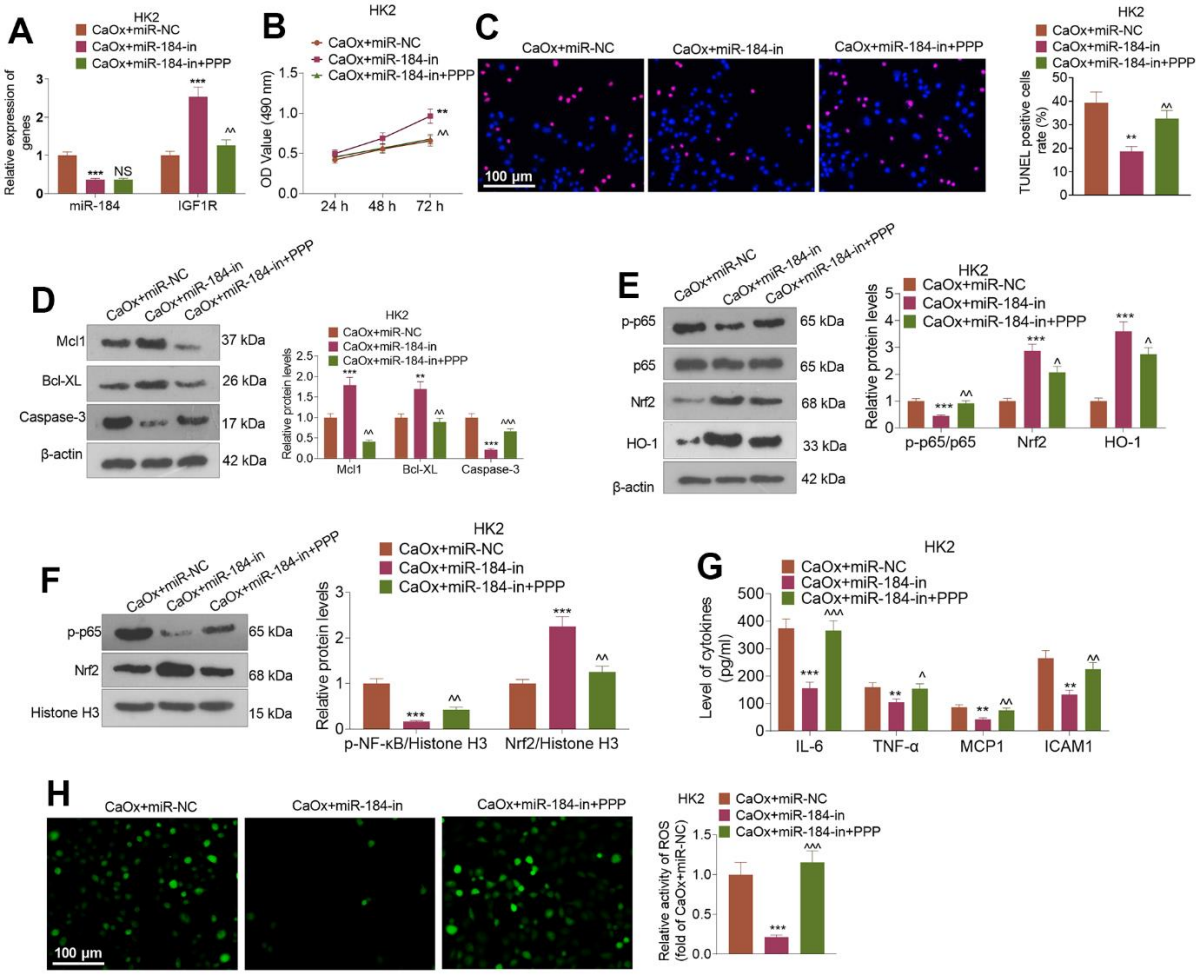
**IGF1R pathway inhibition reversed the inhibitory impact of miR-184 knockdown on renal cell impairment.** Following transfection with miR-184-in and intervention with IGF1R pathway inhibitors (PPP, 1 μMnM), HK2 cells were treated with CaOx (100 μg/mL) for 24 hours. (**A**) The profiles of miR-184 and IGF1R were determined by qRT-PCR. (**B**) A CCK8 assay was performed to examine cell viability. (**C**) Cell apoptosis was monitored through TUNEL staining. Scale bar=100 μm. (**D**) Western blot analysis of the profiles of apoptosis-associated proteins. (**E**, **F**) Western blot analysis verified the profiles of the NF-κB and Nrf2/HO-1 pathways. (**G**) The levels of the inflammatory cytokines IL-6, TNF-α, MCP1, and ICAM1 were determined by ELISA. (**H**) Immunofluorescence evaluated the activity of ROS. Scale bar=100 μm. *P<0.05, **P<0.01, ***P<0.001 (vs. CaOx+miR-in). *nsP>0.05, ^P<0.05, ^^P<0.01* (vs. CaOx+miR-184-in). N=3.

CaOx (100 μg/mL) was applied to treat HK2 cells following the transfection of miR-184-in and intervention with the IGF1R pathway inhibitor (PPP). Figure 7A shows that in contrast with the CaOx+miR-in group, miR-184-in transfection vigorously restrained miR-184 expression and bolstered IGF1R expression. In comparison with the CaOx+miR-184-in group, the use of PPP led to no considerable change in the profile of miR-184 but a stark reduction in that of IGF1R. The CCK8 assay and TUNEL assay revealed that PPP notably repressed HK2 cell viability and boosted cell apoptosis (Figure 7B, 7C). Western blot analysis revealed that PPP administration caused a rise in the levels of Mcl1 and Bcl-XL and a decline in the protein levels of the p-NF-κB and Nrf2/HO-1 pathways (Figure 7D–7F). As suggested by ELISA, PPP remarkably abated the inhibitory effect of miR-184-in on inflammatory factors (IL-6, TNF-α, MCP1, and ICAM1) (Figure 7G). Figure 7H shows that PPP apparently blocked the activity of ROS in HK2 cells.

### Effects of IGF1 and IGF1R inhibitor on miR-184-mediated Rap1 signaling pathway

**Figure 8:**
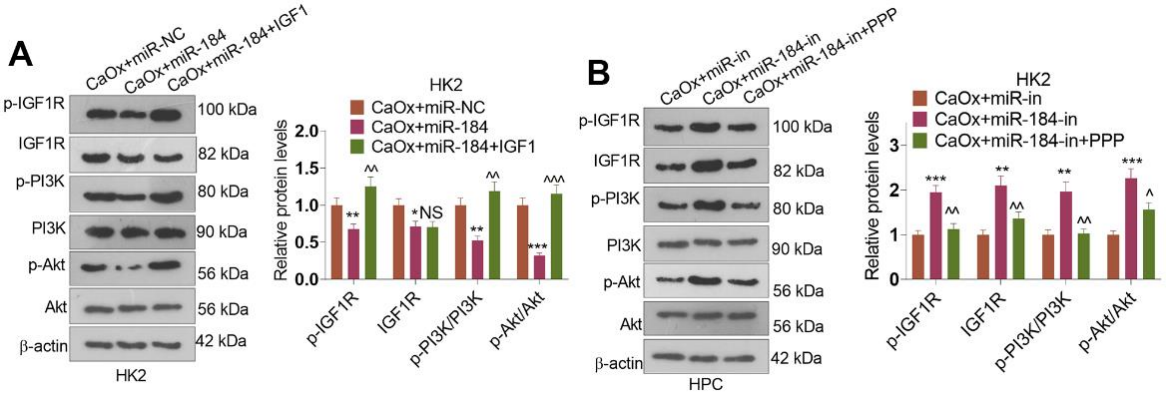
**The alteration of IGF1-PI3K-Akt pathway by miR-184.** CaOx (100 μg/mL) was used to treat HK2 cells transfected with miR-184 mimics or miR-184-in. (**A**, **B**) Western blot was performed to determine p-IGF1R, IGF1R, p-PI3K, PI3K, Akt, and p-Akt in HK2 cells. *P<0.05, **P<0.01, ***P<0.001 (vs. CaOx+miR-in). *NSP>0.05, ^P<0.05, ^^P<0.01, ^^^P<0.001* (vs. CaOx+miR-184 group or CaOx+miR-184-in). N=3.

Western blot was conducted to evaluate IGF1R and PI3K-Akt pathway. These phenomena confirmed that miR-184 mimics repressed p-IGF1R, IGF1R, p-PI3K, and p-Akt in HK2 cells (compared with CaOx+miR-NC group, Figure 8A), while miR-184 inhibition enhanced p-IGF1R, IGF1R, p-PI3K, and p-Akt levels in HK2 cells (Figure 8B). Following IGF1 treatment, p-IGF1R, p-PI3K, and p-Akt levels were al elevated. By contrast, PPP suppressed p-IGF1R, IGF1R, p-PI3K, and p-Akt levels (Figure 8B). Thus, the Rap1 pathway was involved in miR-184-mediated renal cell damage.

## DISCUSSION

The formation of kidney stones involves a complex interplay of various factors. Calcium oxalate is one of the most common components found in kidney stones and is known to contribute to inflammation and oxidative stress in renal cells [19, 20]. Inflammation-induced by calcium oxalate crystals promotes tissue damage, renal cell injury, and recruitment of more inflammatory cells, further exacerbating the inflammatory process [21]. In addition, calcium oxalate crystals can generate ROS directly or indirectly, leading to activation of NADPH oxidase and mitochondrial dysfunction, which finally cause renal cell apoptosis [22]. Repressing the release of pro-inflammatory cytokines, such as IL-1β, TNF-α, IL-6, and oxidative stress mediators, such as ROS, and MDA, can markedly relieve pain and cell apoptosis in kidney stones [23, 24]. Here, we adopted glyoxylate to induce a rat renal calcinosis model and discovered that glyoxylate greatly enhanced miR-184 expression in rat kidney tissues. Further functional assays confirmed that miR-184 inhibition could relieve inflammation, apoptosis, and oxidative stress in the kidney stones cell model, suggesting that miR-184 might be a novel target in kidney stones therapy.

An increasing number of studies have demonstrated that miRNAs are extensively involved in the occurrence and progression of kidney-correlated diseases. For instance, miR-210 intervention suppresses the JAK-STAT pathway to display its anti-inflammatory and anti-apoptotic functions, hence repressing rat renal damage mediated by sepsis [25]. miR-21, upregulated in the mouse renal calcinosis model triggered by glyoxylic acid, has been validated to target PPARA to dampen HK2 cell proliferation and facilitate apoptosis and lipid accumulation [26]. Notably, miR-184 plays a vital role in mediating inflammation and oxidative stress [9, 27]. For example, there are enhanced levels of miR-184 and miR-150 in aging glomerular mesangial cells (GMCs) compared with them in young GMCs. The expression of Rab1a and Rab31 were significantly repressed following miR-184 upregulation, which contributed to lower autophagy activities and higher levels of cellular oxidative products [28]. miR-184's ability to regulate key molecular targets involved in inflammatory pathways and oxidative stress response suggests its potential as a therapeutic target or biomarker for these diseases. Interestingly, we uncovered that calcium oxalate increased the miR-184 profile in HK2 and HPC cells. Further works revealed that calcium oxalate restrained the viability of these cells and increased cell apoptosis, inflammation, and ROS generation, while miR-184 knockdown weakened renal cell injury mediated by calcium oxalate.

The Rap1 signaling pathway plays diverse roles in human diseases, including cancer, cardiovascular diseases, and immune-related disorders [29, 30]. It can promote tumor cell proliferation, invasion, and metastasis. Rap1 activation is associated with increased cell adhesion and migration through its interaction with integrin receptors and modulation of the actin cytoskeleton [31, 32]. The role of the Rap1 signaling pathway in cardiovascular diseases is multifaceted. It participates in endothelial cell function and vascular remodeling. Dysregulated Rap1 signaling can contribute to endothelial dysfunction, leading to impaired vasodilation and increased vascular permeability [33, 34]. Dysregulation of Rap1 can lead to immune-related disorders, including autoimmune diseases and immunodeficiency. In autoimmune diseases, altered Rap1 signaling has been implicated in abnormal immune cell activation, impaired T-cell tolerance, and dysregulated immune cell trafficking [35]. The Rap1 signaling pathway also influences metabolic processes and is implicated in metabolic disorders such as obesity and diabetes. Rap1 has been shown to regulate adipocyte differentiation, energy expenditure, and insulin signaling [36]. Dysregulated Rap1 signaling can disrupt metabolic homeostasis, leading to insulin resistance, impaired glucose metabolism, and dyslipidemia [37, 38]. Recovering the Epac2-Rap1 signaling could mitigate ROS production from mitochondria, and relieve myocardial arrhythmia susceptibility [39]. Epac-Rap signaling pathway has a potent effect on cell-cell and cell-matrix adhesion and maintaining tubular epithelial cell adhesion in renal ischemia Therefore, understanding the functions of the Rap1 signaling pathway in human diseases provides insights into disease mechanisms and potential therapeutic targets.

IGF1 belongs to Rap1 signaling pathway. IGF1 can activate the Rap1 pathway through its receptor, IGF1R. Upon binding of IGF1 to IGF1R, the receptor undergoes autophosphorylation and subsequently activates downstream signaling cascades, including the phosphatidylinositol-3 kinase (PI3K) pathway. Activation of PI3K leads to the activation of various downstream effectors, including the small GTPase Rap1 [40, 41]. In acute renal injury, the activation IGF1-PI3K-Akt pathway has shown prominent protective effects by suppressing apoptosis, inflammation, and oxidative stress [42–45]. Here, in our bioinformatic analysis, we revealed that miR-184 potentially targets IGF1R and is involved in the Rap1 signaling pathway. miR-184 represses the Rap1 pathway by targeting IGF1R. Considering the complex functions of the Rap1 signaling pathway on inflammation, apoptosis, and oxidative stress, it makes sense to regulate Rap1 by mediating miR-184 expression.

However, several shortcomings should be considered in our future studies. First, clinical samples should be collected from patients with kidney stones to uncover the correlation between miR-184 level and clinical features of kidney stones. Second, the protective functions of miR-184 inhibition in the kidney stones murine model should be further confirmed. Based on the evidence, it might be meaningful to develop the diagnostic and treatment strategies for miR-184 in kidney stones in the future.

## CONCLUSIONS

To summarize, this paper has revealed that miR-184 expression is heightened both in a rat renal calcinosis model and in kidney cells treated with calcium oxalate. Further works have shown that miR-184 downregulation activates the Rap1 signaling pathway to ameliorate renal cell inflammation, apoptosis, and oxidative stress mediated by calcium oxalate crystals. This novel miR-184-IGF1R axis may offer reliable references for kidney stones prevention and treatment.

## MATERIALS AND METHODS

### Nephrocalcinosis model

A total of 20 Wistar rats aged 5-6 weeks, with an equal number of males and females, were purchased from Hebei Medical University. Seventeen female and seventeen male rats were chosen at random to form the experimental group. A rat renal calcinosis model was established through the intraperitoneal injection of glyoxylate (80 mg/kg/d; 200 μL). The rest of the animals, intraperitoneally transfused with normal saline of comparable volume, constituted the normal control group. Two weeks later, CO_2_ at a high concentration was applied to euthanize all the rats whose renal tissues were therefore obtained. RNAlater solution (Thermo Fisher Scientific, Shanghai, China) was applied to store the kidney tissues at a low temperature for the following experiments. The research procedures were conducted in accordance with the regulations for the care and use of laboratory animals in the United States.

### Quantitative real-time polymerase chain reaction (qRT-PCR)

qRT-PCR was used to examine the relative profiles of miR-184 and IGF1R in tissues and cells. Based on the sequences of the target genes in GenBank, Primer3.0 software was adopted to design primers that were synthesized by Sangon Biotech (Shanghai, China). Total RNA was extracted from tissues and cells using TRIzol, and its purity and concentration were ascertained by a UV spectrophotometer. miR-184 and IGF1R mRNA were reverse transcribed using the miRNA reverse transcription kit (GeneCopoeia, Guangzhou, China) and ReverTra Ace qPCR RT kit (Toyobo, Osaka, Japan), respectively, to obtain the first strand of synthesized cDNA. With the strand as the template, the miRNA amplification kit (Wuhan Pure Biotechnology Co., Ltd., Wuhan, China) and SYBR Green qPCR Master Mix (MedChemExpress, NJ, USA) were deployed to amplify the target genes. U6 was used as the internal reference for miR-184, and GAPDH was used as the internal reference for IGF1R. The relative profiles of miR-184 and IGF1R were calculated in accordance with the 2^-∆∆CT^ formula. The primer sequences are detailed in the table below:

**Table:** 

**Genes (Rat)**	**Primer sequences**
miR-184	F: GCCGAGTGGACGGAGACTGAUAAGT
	R: CAGTGCGTGTCGTGGAGT
U6	F: TGCGGGTGCTCGCTTCGGCAGC
	R: CCAGTGCAGGGTCCGAGGT
IGF1R	F: TCCCAAGCTGTGTGTCTCTG
	R: GTGCCACGTTATGATGATGC
GAPDH	F: TGACTGTGCCGTTGAACTTG
	R: GAGACAGCCGCATCTTCTTG

**Table:** 

**Genes (Human)**	**Primer sequences**
miR-184	F: GGTGGACGGAGAACTGAT
	R: GAGGAGGAAGAAGGGTAGGA
U6	F: CGCTAGCACATATCGGCTA
	R: TTCTGCGACGAATTTGTCAT
IGF1R	F: AAAGAATTCAGTGTGTGGCGGCGGCGG
	R: AAAGTCGACTCCTTTTATTTGGGACGA
GAPDH	F: AGGTCGGTGTGAACGGATTTG
	R: TGTAGACCATGTAGTTGAGGTCA

### Culture and transfection of cells

The Chinese Academy of Sciences (Shanghai, China) supplied the HK2 and human podocyte line (HPC) cells utilized in the research. The cells were cultivated with DMEM high glucose culture solution (Thermo Fisher Scientific, Shanghai, China) incorporating 10% fetal bovine serum and 1% penicillin/streptomycin in an incubator with 5% CO_2_ and saturated humidity at 37° C. The experiments were conducted on cells in the logarithmic growth phase. GenePharma (Shanghai, China) was responsible for the construction of miR-184 mimics and miRNA control (miR-NC), the miR-184 inhibitor (miR-184-in) and miR-in. HK2 and HPC cells were transfected with Lipofectamine® 3000 (Invitrogen; Thermo Fisher Scientific) as instructed by the supplier. The efficacy of transfection was assessed by qRT-PCR 48 hours later. HK2 cells and HPCs, before or after transfection, were treated with calcium oxalate (5, 10, 25, 50, 100, or 200 μg/mL) to establish the *in vitro* cell experiment model. For IGF1R inhibition or activation, the IGF1R inhibitor picropodophyllin (PPP) (1 μM; Santa Cruz Biotechnology, Paso Robles, CA, USA) and IGF1R activator IGF-1 (100 ng/ml; ProSpec, Israel) were administered into the culture medium for 1 h.

### Cell-counting kit 8 (CCK8) assay

HK2 and HPC cells subjected to various stimuli were plated in 96-well plates. Following 24, 48, and 72 hours of incubation, 10 μL of CCK-8 solution (Beyotime Biotechnology, Shanghai, China) was added to each well. The incubator was adopted for another 4 hours of culture, after which a microplate reader (Thermo Fisher Scientific, Shanghai, China) examined the absorbance of each well (490 nm).

### Terminal deoxynucleotidyl transferase dUTP nick end labeling (TUNEL) staining

PBS was given to rinse HK2 and HPC cells that were treated with different factors. 4% paraformaldehyde was used to fix the cells for 30 minutes, which were then washed in PBS once. With the addition of the immunostaining detergent, the cells were incubated in an ice bath for 2 minutes. Fifty microliters of TUNEL detection solution (Boster Bioengineering Co., Ltd., Wuhan, China) was applied to the samples for 60 minutes of incubation in darkness at room temperature (RT). After being rinsed in PBS three times, the cells were stained by 4',6-diamidino-2-phenylindole (DAPI) solution (Beyotime, Shanghai, China) for nuclear staining. Finally, a microscope was used to observe TUNEL-positive cells (labeled with red fluorescence). Five nonoverlapping fields were chosen at random from each sample for counting. The TUNEL-positive cell rate was counted as the ratio of TUNEL-positive cells to DAPI-positive cells.

### Immunofluorescence

Following cell treatment, 4% paraformaldehyde (soluble in PBS, pH 7.4) was used for cell fixation for 10 minutes. The cells were washed 3 times with ice PBS, then incubated with PBS containing 0.1-0.25% Triton X-100 for 10 minutes. Cells were incubated with PBST (PBS+0.1% Tween 20) containing 1% BSA and 22.52 mg/mL glycine for 30 minutes for blocking antibodies. The primary antibody anti-IGF1R (1:200, ab182408) was added and incubated with the cells at 4° C for 15 hours. The cells were washed with PBS three times for 5 minutes each time, and incubates cells with Goat anti-rabbit IgG (Alexa Fluor® 488) (1:500, ab150077) (soluble in 1% BSA) in dark for 1 hour. After washing the cells with PBS in the dark for 3 times, DAPI was used for nuclear staining. A microscope was used to observe IGF1R-positive cells.

### Western blot

Radioimmunoprecipitation assay (RIPA) lysis buffer was used to extract total protein from HK2 cells and HPCs. 25 μg of the total protein was utilized for polyacrylamide gel electrophoresis and then transferred onto a membrane at 100 V for an hour. After being blocked with 5% skim milk powder (RT, 1 hour), the membrane was flushed with TBST and then incubated with the following primary antibodies: anti-Mcl1 (1:2000, ab32087), anti-Bcl-XL (1:1000, ab223547), anti-caspase-3 (1:2000, ab184787), anti-IGF1R (1:1000, ab182408), anti-IGF1R (phospho Y1161) antibody (1:1000, ab39398), anti-PI3K (1:1000, ab191606), anti-PI3K (phospho Y607) antibody (1:1500, ab182651), anti-Akt (1:2000, ab8805), anti-AKT (phospho T308) antibody (1:100, ab38449), anti-NF-κB p65 (1:2000, ab32536), anti-p-NF-κB p65 (1:1000, ab239882), anti-Nrf2 (1:500, ab137550), anti-HO-1 (1:10000, ab68477), anti-histone H3 (1:1000, ab1791), and anti-β The next morning, the membrane was incubated with horseradish peroxidase (HRP)-labeled secondary antibody (1:3000, ab6721) at RT for 30 minutes. Following TBST washing, an ECL kit (Amersham Pharmacia Biotech, Little Chalfont, UK) was employed for development, and ImageJ was utilized to determine the gray value of each protein. All the antibodies mentioned above were acquired from Abcam (Shanghai, China).

### Enzyme-linked immunosorbent assay (ELISA)

Stably transfected HK2 and HPC cells were inoculated into a 6-well plate in the logarithmic growth phase. Twenty-four hours after drug treatment, fresh culture medium was added for another 48 hours of culture. Trypsin was applied to digest and centrifuge the cells for 10 minutes at 1000×g to obtain the cell supernatant. Corresponding ELISA kits (Abcam, Shanghai, China) were used to confirm the contents of the cytokines, including interleukin (IL)-6, tumor necrosis factor-alpha (TNF-α), monocyte chemoattractant protein 1 (MCP1), and intercellular adhesion molecule 1 (ICAM1) in line with the supplier’s recommendations.

### Reactive oxygen species (ROS) activity detection

The activity of ROS in HK2 cells and HPCs was determined by the Reactive Oxygen Species Assay Kit (Yisheng Biotechnology, Shanghai, China). HK2 and HPC cells post transfection, seeded into 96-well plates (1×105 cells/well), were cultivated for 24 hours following treatment with drugs. DCFH-DA (10 μL) was added to each well for 30 minutes of incubation in darkness at room temperature. Finally, a fluorescence microscope was used to examine the fluorescence intensity, thus reflecting the activity of ROS.

### Dual luciferase assay

Promega (Promega, Madison, WI, USA) established luciferase reporter vectors (IGF1R-WT and IGF1R-mut). HK2 and HPC cells were seeded into 48-well plates at a density of 4.5×10^4^/well. When the cells achieved 70% confluence, these vectors were transfected, along with miR-184 mimics or miR-184-NC, into HK2 and HPC cells by applying Lipofectamine® 3000 (Invitrogen; Thermo Fisher Scientific). The luciferase activity of each well was assessed 48 hours later using the Dual-Luciferase Reporter Assay System (Promega).

### RNA-immunoprecipitation (RIP)

RIP analysis was performed using the Magna RIP Kit (Millipore, Bedford, MA, USA). When HK2 and HPC cells reached over 80% confluence, RIP lysis buffer was applied to lyse the cells. The whole cell lysates were conflated with RIP buffers incorporating magnetic beads combined with the human anti-Ago2 antibody and negative control normal mouse IgG antibody (Millipore). Protease K was used to digest the proteins and isolate the RNA. RNA purity and concentration were measured by an ultramicro spectrophotometer (Thermo Fisher Scientific, Shanghai, China). qRT-PCR confirmed the profile of the RNA.

### Analysis of statistics

All statistical analyses in the study were performed using GraphPad Prism 8 (GraphPad Software, San Diego, CA, USA). Measurement data are displayed as the mean ± standard deviation (x ± s). As Student’s *t*-test was used to compare two different groups, one-way ANOVA was employed for comparisons among multiple groups with the Tukey post hoc test. The correlation between miR-184 and IGF1R was evaluated using Pearson correlation analysis. *P<0.05* was considered to indicate statistical significance.

### Data availability statement

The data sets used and analyzed during the current study are available from the corresponding author upon reasonable request.
